# Yolk sac development in dogs: from morphology to functional aspects

**DOI:** 10.1590/1984-3143-AR2025-0104

**Published:** 2026-02-27

**Authors:** Caio Garcia Barbosa da Silva, Julia Rodrigues Greghi, Maria Fernanda Frasson Pontes, Guilherme Schiess Cardoso, Maria Isabel Mello Martins, Carlos Eduardo Ambrósio, Phelipe Oliveira Favaron

**Affiliations:** 1 Departamento de Clínicas Veterinárias, Centro de Ciências Agrárias, Universidade Estadual de Londrina – UEL, Londrina, PR, Brasil; 2 Departamento de Biologia Geral, Centro de Ciências Biológicas, Universidade Estadual de Londrina – UEL, Londrina, PR, Brasil; 3 Departamento de Medicina Veterinária, Faculdade de Zootecnia e Engenharia de Alimentos, Universidade de São Paulo – USP, Pirassununga, SP, Brasil

**Keywords:** extraembryonic membranes, stem cells, hematopoiesis, development

## Abstract

The yolk sac (YS) is a multifunctional and essential structure for early maternal-fetal interactions, playing roles in nutrient supply, organogenesis, immune modulation, and hematopoiesis. Unlike most species, dogs maintain the YS until the end of pregnancy, yet little is known about its functional activity, particularly in later stages. Animal models are crucial for exploring maternal-fetal processes and offering insights into comparative development, including in humans. This study aimed to analyze the YS structure in canine embryos and fetuses at early (up to 20 days, n = 6), middle (21–40 days, n = 6), and late (41–60 days, n = 6) stages. Gestational age was estimated using crown-rump length (CRL) and external anatomy. Samples from the university’s Veterinary Hospital and neutering campaigns in Londrina (Paraná, Brazil) were fixed in 10% formaldehyde for morphological analysis (H&E, PAS, and Picrosirius red) or 4% paraformaldehyde for OCT4 immunostaining (CEUA/UEL protocol number 029.2022). Histologically, the YS showed a trilaminar structure with blood islands in the intermediate layer surrounded by mesenchyme. Within these islands, undifferentiated cellular niches (OCT4+) were identified. During gestation, the YS changed from a thin, smooth, pale-red membrane to a wrinkled structure with narrowed ends and a thickened central region, resembling a "whale tail." The red color, indicative of vascularization, intensified with blood island enlargement. Collagen secretion, particularly type I, progressively increased around the blood islands. Glycoprotein deposits persisted until late gestation, as shown by PAS staining. These findings highlight the structural, secretory, and reserve roles of the canine YS throughout pregnancy.

## Introduction

The four extraembryonic membranes—chorion, amnion, allantois, and yolk sac—act in a coordinated manner to support embryonic development in Eutheria, each contributing distinct structural and functional roles throughout gestation (Carter, 2024; Laundon et al., 2024). Among them, the yolk sac stands out for its functional (Dzierzak and Bigas, 2018), molecular (Ross and Boroviak, 2020), and therapeutic relevance (Pinto et al., 2021). Its persistence and role during gestation vary among species. In pigs and ruminants, it is transient and does not contribute to definitive placentation (Bertassoli et al., 2015; Galdos-Riveros et al., 2015). In horses, it serves initially in the formation of the temporary choriovitelline placenta, later regressing (Ginther, 2022). In contrast, in rodents and lagomorphs, it persists until birth, forming the yolk placenta alongside the chorioallantoic placenta, with well-documented developmental and functional roles (Vale et al., 2013; Oliveira et al., 2012; Ornoy and Miller, 2023).

In carnivores, data on yolk sac morphology are scarce and limited to specific gestational stages (Table 1). In early canine pregnancy, the yolk sac forms the transient choriovitelline placenta directly participating in maternal–embryonic exchanges. As gestation progresses and the definitive endotheliochorial allantochorionic placenta becomes established, the yolk sac gradually loses its placental function (Carter and Mess, 2017; Kowalewski et al., 2021; Corte et al., 2025). However, unlike in many other domestic mammals, the yolk sac does not regress in carnivores; classical studies demonstrated its persistence throughout gestation (Tiedemann, 1976; Tiedemann, 1977; Lee et al., 1983). At term, it remains as a red, inverted T-shaped membrane attached to the fetus, with elongated projections that persist until birth (Miglino et al., 2006). In both dogs and cats, the yolk sac continues to play an essential metabolic role late in gestation, particularly in serum protein biosynthesis before liver maturation, thereby contributing to fetal development (Tiedemann and Minuth, 1980; Miglino et al., 2006).

**Table 1:** Morphological Studies of the Yolk Sac in Carnivores.

**Days of gestation**	**Macroscopy**	**Techniques used**	**Species**	**Characteristics**	**References**
10,14,18,57	Yes	H&E	*Canis familiaris*	Initially thin (endoderm + primitive vessels); progressive thickening with connective tissue deposition. Becomes more robust near term.	Aralla et al. (2013)
20,24,30,45	Yes	H&E, Picrosirius, SEM	*Canis familiaris* and *Felis catus*	Large and vascularized (inverted T-shape); connected to the umbilical cord. Presence of hemangioblasts. Functional until mid-gestation.	Miglino et al. (2006)
40,50,60	No	TEM	*Canis familiaris*	High early synthetic activity (mitochondria, ribosomes); progressive degeneration. Erythropoiesis active until ~day 50. Strong vasculature and vesicular transport.	Lee et al. (1983)
~37–53	Yes	H&E, IHC, TEM, SEM, Perl’s, Masson's	*Nasua nasua*	Initially avascular; later vascularized and specialized (iron accumulation). Functionally active until late gestation.	Favaron et al. (2014)
~21- 40	Yes	H&E, Masson, PAS, Acid fuchsin, Iron hematoxylin	*Ursus americanus*	Prominent early on (bilaminar wall, vitelline vessels); later elongated and attached mesometrially. Hematopoietic, with absorptive and secretory roles.	Wimsatt (1974)
14-66	No	Histochemistry, Polychrome staining, Methylene blue, TEM	*Felis catus*	High metabolic activity (endoderm/mesothelium); mesenchyme with blood islands. Increasing vascular complexity. Active until late gestation, even without yolk.	Tiedemann (1976, 1977)

H&E: hematoxylin and eosin; PAS: periodic acid–Schiff; IHC: immunohistochemistry; TEM: transmission electron microscopy; SEM: scanning electron microscopy.

The diverse morphofunctional characteristics of the yolk sac have been explored in several scientific fields, contributing to basic knowledge in Developmental Biology (Galdos-Riveros et al., 2015; Matias et al., 2022a), advancements in assisted reproduction techniques such as bovine cloning (Alberto et al., 2013; Maiorka et al., 2015; Mess et al., 2017), and applications in Regenerative Medicine (Anunciação et al., 2017; Fratini et al., 2018; Matias et al., 2018). In carnivores—particularly dogs—there is growing interest in the yolk sac as a source of stem cells (Sousa et al., 2021; Pereira et al., 2023; Motta et al., 2023). It has been identified as a promising reservoir of mesenchymal (Wenceslau et al., 2011) and hematopoietic stem cells (Sousa et al., 2021), which can differentiate into endothelial cells after VEGF stimulation (Fratini et al., 2016) and form intestinal organoids (Motta et al., 2023). These cells have also been applied in experimental therapies, such as treatment of canine hip dysplasia (Benedetti et al., 2019), and in tissue engineering, including the recellularization of canine trachea scaffolds (Matias et al., 2022b).

Despite recent advances in isolating and cultivating yolk sac-derived stem cells in dogs, there is still a lack of detailed information regarding the morphology and structural changes of this membrane during gestation. Unlike in ruminants and primates, the yolk sac of carnivores remains vascularized and functional until birth. Understanding its development is therefore essential to clarify the reasons for its persistence and how structural changes relate to its cellular composition and functions. Thus, this study aimed to perform a detailed morphological analysis of the canine yolk sac at different gestational stages—early (≤20 days), mid (21–40 days), and late (41–60 days)—to characterize its macroscopic and microscopic features throughout development.

## Methods

### Macroscopic analysis

Canine embryos and fetuses were obtained at early (Group I: up to 20 days, n = 6), middle (Group II: 21–40 days, n = 6), and late (Group III: 41–60 days, n = 6) gestational stages, collected from pregnant bitches whose pregnancies were identified by chance during spay campaigns in Londrina (Paraná, Brazil) or from cesarean sections performed at the university’s Veterinary Hospital. After collection, uteruses were fixed in 10% formaldehyde or 4% paraformaldehyde and sent to the Extracellular Matrix Laboratory, where the gestational sacs were dissected to isolate the embryos/fetuses.

In spay campaigns with high demand, pregnancies were not previously diagnosed and samples were collected opportunistically; therefore, it was not possible to determine the exact gestational age at the time of collection or to obtain important information about the donor bitches, such as breed, age, body size, or reproductive history. As the vast majority of samples originated from spay campaigns, the method for gestational age estimation was standardized across all samples. This estimation was performed by measuring the crown–rump length (CRL) using a digital caliper, according to Evans and Sack (1973), and confirmed through external morphological analysis (Evans and Sack, 1973; Pieri et al., 2015). All procedures were approved by the institutional Ethics Committee (protocol nº 029.2022).

### Histological analysis

After isolation, yolk sac and embryo samples were fixed, dehydrated in increasing ethanol concentrations, diaphanized in xylene, and embedded in Paraplast (Oxford Lab., USA), as described by Favaron et al. (2014). Sections of 5 μm were obtained using an automatic microtome (RM2155; Leica, Germany) and stained with hematoxylin-eosin (HE), picrosirius-hematoxylin, Masson's trichrome, and periodic acid-Schiff (PAS), according to protocols described by Tolosa et al. (2003). The slides were analyzed using an Olympus BX40 light microscope coupled with a Zeiss KS400 image analysis system.

### Immunohistochemical analysis

Immunohistochemistry was performed following methodological principles adapted from Oliveira et al. (2012) and Favaron et al. (2016), Tissues were fixed in 10% neutral-buffered formalin for 24 h, processed routinely, embedded in paraffin, and sectioned at 3–5 µm. Sections were deparaffinized and rehydrated, and endogenous peroxidase activity was blocked with 3% hydrogen peroxide for 20 min. Antigen retrieval was conducted in 10 mM citrate buffer (pH 6.0) using microwave heating at 750 W for 15 min, followed by passive cooling at room temperature. Non-specific binding was blocked with Dako Protein Block for 20 min. The primary antibody anti-OCT4 (sc-4420, Santa Cruz Biotechnology; 1:100 in PBS with 1% BSA) was applied overnight at 4 °C in a humid chamber. After PBS washes, the biotinylated secondary antibody and streptavidin-HRP (LSAB System-HRP, Dako) were incubated at room temperature for 45 min each. Visualization was achieved using DAB chromogen for 3 min under microscopic monitoring, followed by hematoxylin counterstaining and permanent mounting. Negative controls were performed by replacing the primary antibody with isotype IgG (Goat anti-Mouse IgG – AP308F, Chemical International), processed under identical conditions to assess nonspecific background staining.

## Results

**Figure 1:**
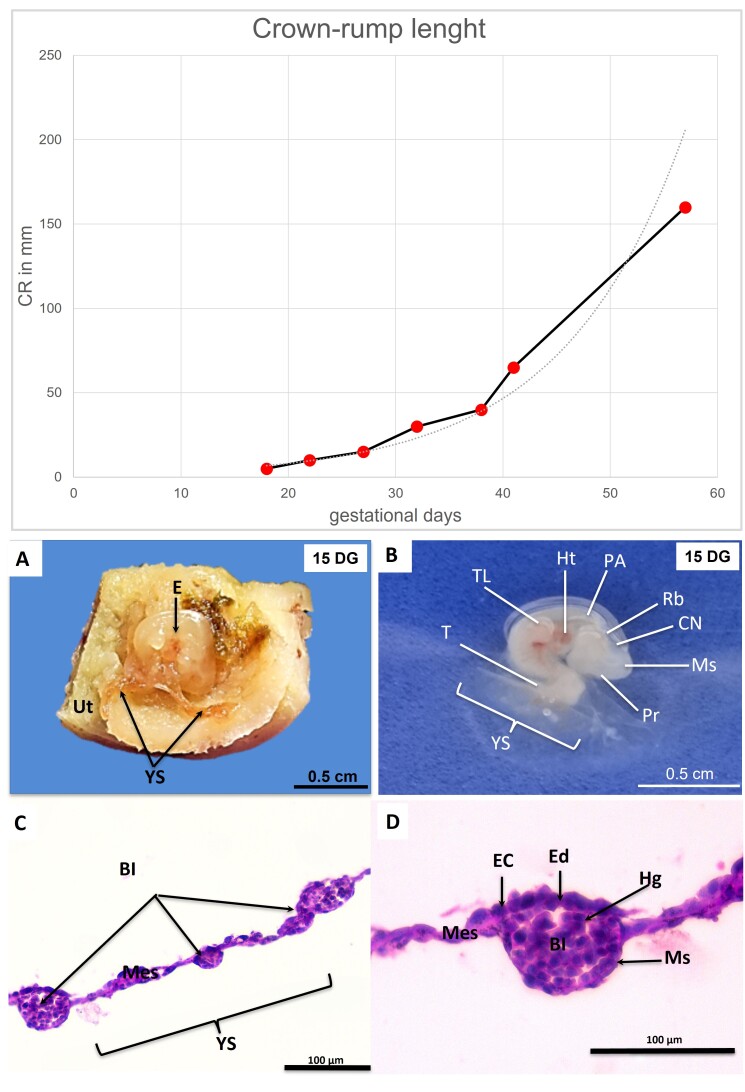
**(A)** Crown-rump length (CRL) curve of canine embryos and fetuses correlated with gestational age. **(B,C)** Photographs of an 18-day embryo. **(B)** Topographic view of the yolk sac (YS) and its extremities (arrows) relative to the uterus (Ut). E = embryo. **(C)** After removal from the uterus, visible structures: mesencephalon (Ms), prosencephalon (Pr), rhombencephalon (Rb), pharyngeal arches (PA), heart (Ht), thoracic limb (TL), cranial neuropore (CN), tail (T), and yolk sac (YS). **(D,E)** Photomicrographs of the yolk sac. **(D)** Established vascularization (blood islands = BI) in the mesenchyme (Mes). **(E)** Hemangioblasts (Hg) inside BI. Note: endodermal cells (EC), endothelium (En), mesothelium (Ms), mesenchyme (Mes). Hematoxylin-Eosin staining.

After obtaining the CRL and analyzing the external morphological characteristics, the gestational age of each embryo and fetus was estimated, as illustrated in Figure 1A. Based on this, the samples could be divided into three groups: early (up to gestational day 20), mid (21–40 days of development) and late (41–60 days) gestation. In Figure 1A, the red dots indicate the respective ages of the embryos and fetuses used in the research. For each group, the macroscopic descriptions of the embryos or fetuses, the yolk sac and the respective microscopic descriptions are presented respectively.

### Early pregnancy

In this group, the embryos had an estimated gestational age of 18 days, according to the methodology of Evans and Sack (1973), with a CRL of 5 mm and corresponding external characteristics, such as the C-shaped body curvature, the presence of pharyngeal arches and optic vesicles, and the open cranial neuropore, all consistent with this developmental stage (Figures 1B and 1C). The yolk sac appeared as a thin membrane, light red in color and smooth in appearance. As for its distribution, it was limited to the central region of the embryo, where it connects to the primitive intestine. In the histology of the yolk sac, a trilaminar structure was observed. Facing the coelomic cavity, there is the endoderm, which appears with round and spaced cells (Figures 1D and 1E). Facing the vitelline cavity, the mesothelium, consisting of squamous cells, was identified. In the intermediate layer, there is the mesenchyme, composed of an extracellular matrix and with little cellular content. Lodged in the mesenchyme are the blood islets, oval structures that contain hemangioblasts, the precursors of all the cells of the blood lineage. These cells have a rounded shape, with a developed, centralized nucleus and little cytoplasmic content (Figure 1E).

### Mid-gestation

**Figure 2:**
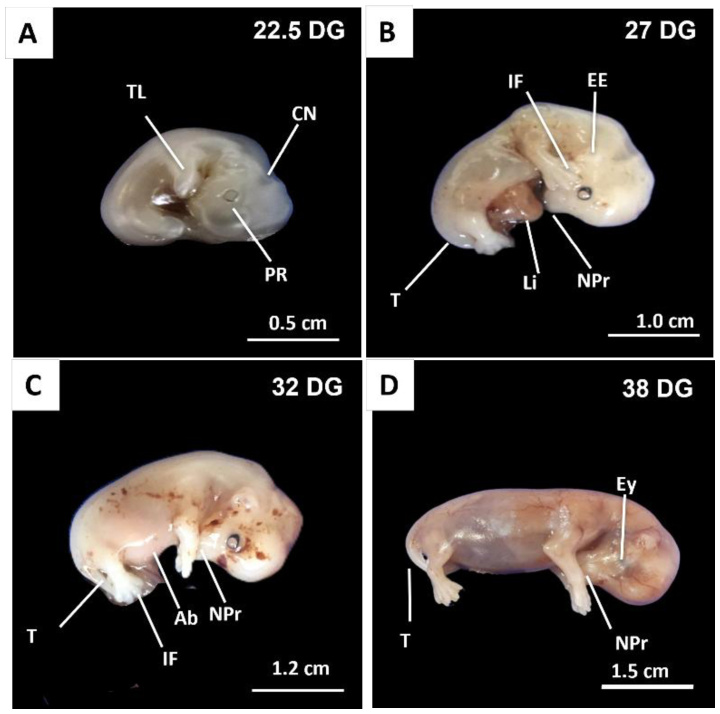
Photographs of canine embryos and fetuses at 22, 27, 32 and 38 days of gestation. Externally, the following structures and regions are observed: buds of the thoracic (TL) and pelvic (PL) limbs, cranial neuropore (CN), optic vesicle with pigmented retina (PR), tail (T), liver (Li), external ear (EE), interdigital fissure (IF), eyelid (Ey); abdomen (Ab) and nasal process (NPr).

In this group, embryos estimated at 22, 27, 32, and 38 days of gestation were examined to enable macroscopic comparison across the developmental continuum. At 22 days (CRL 10 mm), embryos remained C-shaped, with an open cranial neuropore, early retinal pigmentation, and initial definition of the thoracic limb buds (Figure 2A). By 27 days (CRL 15 mm), ocular pigmentation had intensified, interdigital grooves had begun to form, the cranial neuropore was closed, and external genital differentiation had become visible (Figure 2B). At 32 days, the body axis had elongated, the abdominal wall was closed, and interdigital grooves had deepened (Figure 2C). By 38 days, fetuses exhibited eyelid coverage, developed external genitalia, and a more advanced auricular structure (Figure 2D).

**Figure 3:**
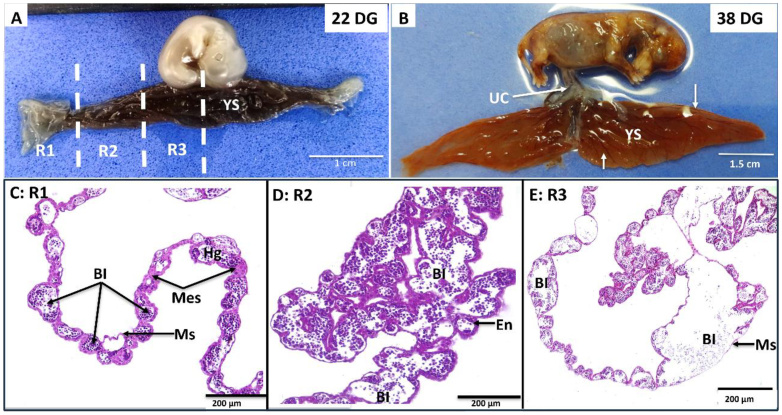
In **(A)** and **(B)**: Photographs of canine embryo and fetus at 22 and 38 days of gestation, respectively. Macroscopically, the yolk sac (YS) has an elongated shape with two lateral projections. Structurally it was divided into regions 1 (R1), 2 (R2) and 3 (R3), according to the insertion of its central part into the umbilical cord (UC). At 38 days, the yolk sac acquires the shape of a “whale tail” with blood vessels (arrows) distributed throughout its length. **(CD)**: Photomicrographs comparing the histology of the three regions of the yolk sac at mid-gestation (32 days of development). Note the gradual increase in the distribution and volume of blood islands (BI), containing hemangioblasts (Hg) inside. Note: endothelium (En), mesenchyme (Mes) and mesothelium (Ms). Hematoxylin-Eosin staining.

For this developmental stage, the yolk sac was divided into three regions for a detailed and comparative analysis. Region 1 corresponds to the end of the yolk sac, where it connects to the amniotic membrane; region 2, to the intermediate region; and region 3, to the site of greatest thickening, where the yolk sac connects to the primitive intestine (Figure 3A). Macroscopically, the yolk sac acquired a wrinkled appearance and an intense red color, due to the increase in its vascularization (Figure 3B). In addition, the two ends became narrower and the central region thickened, so that the yolk sac began to be shaped as an inverted “T”, or “whale tail” (Figure 3B).

Region 1**:** The yolk sac showed numerous small-diameter blood islets; some were filled with hemangioblasts, while others appeared empty. The endodermal cells displayed a characteristic rounded shape, resting on the mesenchyme. In front of the endoderm, mesothelial cells with flattened nuclei were observed (Figure 3C).

Region 2**:** The development of blood islands was more pronounced, surpassing the previous region in both quantity and diameter. Large blood islets were noted, some of which were partially filled with blood lineage cells. Additionally, the mesenchymal layer is thicker, allowing the membrane to accommodate a greater number of vessels (Figure 3D).

Region 3**:** The histological structure resembled that of Region 2; however, large vessels surrounded by extensive mesenchymal content were observed, along with a more developed network of villi overlapping one another, indicating a substantial blood supply to the region (Figure 3E).

**Figure 4:**
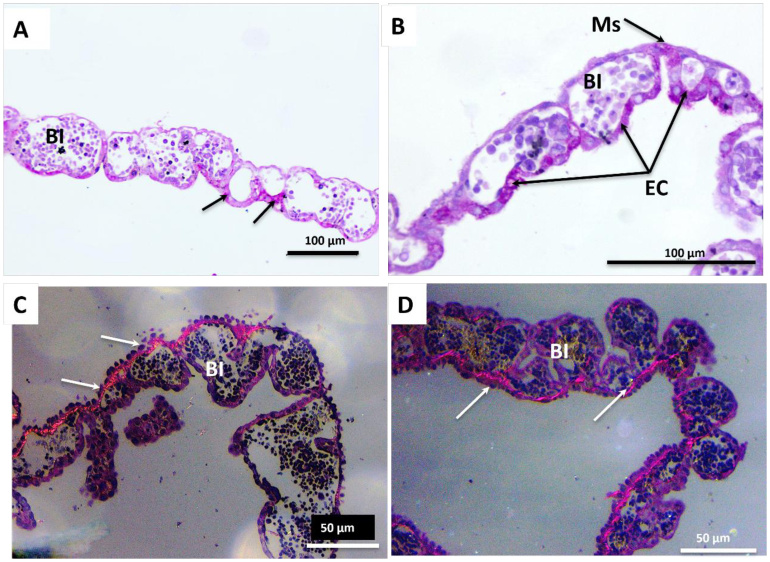
Photomicrographs of the yolk sac in mid-gestation (32 days) of region 3. **(A)** and **(B)**: Note the glycogen deposition in the cytoplasm of the endodermal cells (EC) and in the mesothelium (Ms) region surrounding the blood islands (BI). Staining: periodic acid-Schiff (PAS). In **(C and D):** Bundles of type I collagen fibers stained in red (arrows), surrounding the wall of the blood islands (BI). **(C and D)**: Picrosirius red staining analyzed under polarized light.

In PAS staining, glycogen deposition was identified, appearing dark purple, surrounding the blood islands, more specifically in the regions encircling the endodermal cells. In region 3 (central), a greater quantity and intensity of glycogen deposition areas were observed (Figures 4A and 4B). Using Picrosirius staining, bundles of collagen fibers were noted around the blood islands. In all regions, a subtle presence of type I fibers, stained red-orange (Figure 4C and 4D), was observed.

### End of gestation

**Figure 5:**
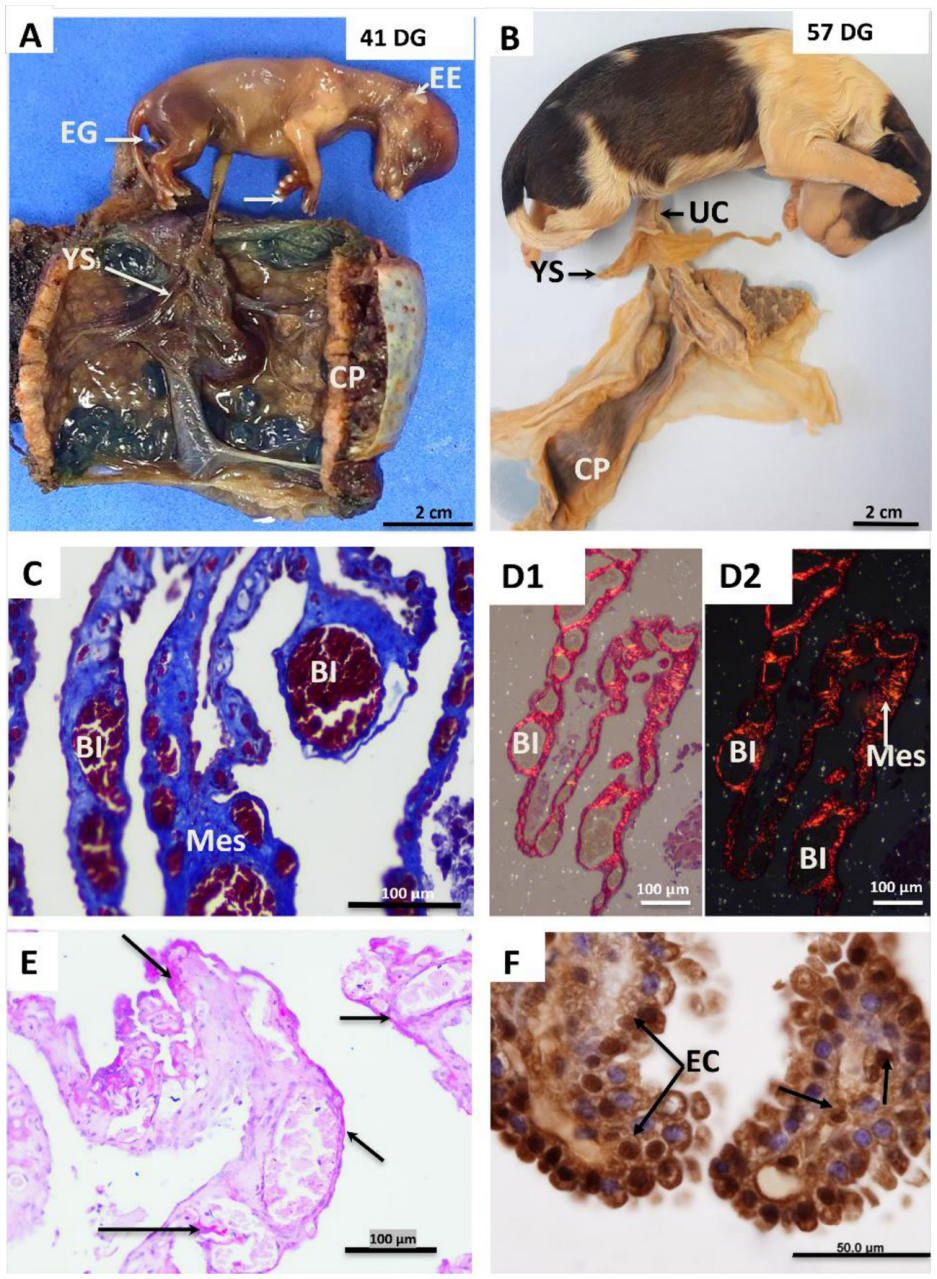
In **(A)** and **(B)**: Photographs of fetuses and canine yolk sac at the end of gestation. **(A)** 41 days gestation **(B)** 57 days gestation. Note the arrangement of the yolk sac (YS) in relation to the chorioallantoic placenta (CP) and its insertion next to the umbilical cord (UC) In fetuses, it is also possible to see the external ear (EE), external genitalia (EG), nails (arrow) and hair covering the body of the fetus at 57 days of gestation. **(C)** Histology of the yolk sac at 57 days of gestation stained using the Masson's Trichrome technique. Note areas of collagen deposition (total) stained in blue around the wall of the blood islands (BI) and in the mesenchyme layer (Mes). **(D1)** and **(D2):** Picrosirius red staining, without and with polarization, respectively. Note the abundance of type I collagen fibers (red), long and thick, distributed mainly around the wall of the blood islands (BI) and in the mesenchymal layer (Mes). In **(E):** Glycogen deposits (arrows) in the cytoplasm of the endodermal cells (EC) of the yolk sac. In **(F):** Immunostaining for OCT4, indicating the presence of pluripotent cells in the yolk sac endoderm (En) and inside the blood islands (arrows).

In this group, fetuses at 41 and 57 days of gestation (term) were analyzed. Overall, the 41-day-old fetuses exhibited a significant body growth, with further development of the thoracic and pelvic limbs, external ear, footpads, and nails (Figure 5A). Additionally, the initial formation of tactile hairs was observed. The term fetus (57 days) had all structures fully formed, with consolidated and pigmented skin tissue and body hair (Figure 5B). The yolk sac persisted until the end of gestation, maintaining an inverted "T" shape, villous structure, and intense red coloration, reaching up to 12 centimeters in length. Histologically, the number of villi increased proportionally to the growth in both quantity and diameter of the blood islands. Using Masson's trichrome staining, total collagen (stained blue) was observed throughout the structure of the yolk sac, with greater intensity around the blood islands (Figure 5C). In corroboration, Picrosirius red staining characterized numerous collagen fibers surrounding the walls of the blood islands, predominantly type I collagen (stained red), formed by longer and thicker fibers (Figure 5D). Additionally, intense glycogen deposition was observed around the endodermal cells around the blood islets (Figure 5E). The presence of Oct-4 positive cells was also observed at this gestational stage. These cells were present both in the endodermal layer of the yolk sac and within the blood islets (Figure 5F).

## Discussion

Because the collected pregnant uteruses lacked reproductive history, gestational age was estimated using the crown–rump length (CRL) method described by Evans and Sack (1973). Overall, the developmental sequence observed in our specimens was consistent with the classical CRL methodology, demonstrating conformity with the expected progression of external morphological features. However, some small variations were noted, such as: (1) the appearance of external genitalia at 27 days instead of the expected 30 days, (2) closure of the abdominal wall at 32 days rather than around 40 days, and (3) eyelid fusion also at 38 days instead of the anticipated 35 days. These discrepancies were expected, as the original CRL parameters were established in Beagle dogs, whereas mixed-breed dogs display greater morphological variability. Importantly, previous studies using mixed-breed conceptuses (Pieri et al., 2015; Casals et al., 2020; Scassiotti et al., 2023) likewise confirmed that the overall developmental pattern remains aligned with the framework proposed by Evans and Sack. Nonetheless, the CRL method provides only an estimated gestational age and therefore represents an inherent limitation of this study, together with the modest sample size per developmental phase. Even so, it remains a valuable tool for comparative embryological analyses when samples are obtained under opportunistic collection conditions.

In many species, the yolk sac is a transient structure with no role in placental formation, though its regression varies: it is absorbed by the end of the first third of gestation in pigs (Bertassoli et al., 2015), between 40–60 days in ruminants (Mess et al., 2017), and after initial choriovitelline placental formation in horses (Ginther, 2022). In contrast, in carnivores the yolk sac persists until the end of gestation (Miglino et al., 2006; Favaron et al., 2014), as also observed in the present study. These observations raise questions about the continued functionality of the yolk sac to assist in the nutrition of the embryo and fetus throughout gestation, as well as the fact that the yolk sac represents an active site of hematopoietic stem cell production.

In carnivores, initial placentation is marked by the presence of a transient choriovitelline placenta, which forms when the vascularized part of the yolk sac comes into direct contact with the trophoblast (Carter and Mess, 2017). As this interface detaches—due to the expansion of the allantois and exocoelomic growth—the yolk sac ceases to function as a placenta, transforming into a distinct and prominent structure in the extraembryonic cavity (Lee et al., 1983). In our research, this change was already perceptible on the 18th day of gestation, when the choriovitelline placenta was no longer identifiable and the yolk sac emerged as an autonomous structure, distinct from the definitive placenta. These results corroborate with classic research, such as that of Tiedemann (1976), who reported the functional replacement of the choriovitelline placenta by the chorioallantoic placenta approximately on the 20th day of gestation in cats.

In addition to these characteristics described by the authors, it was found that the yolk sac presents several variations in its morphological structure, which are reflected in its support, hematopoietic and reserve functions during gestation in dogs. Approximately on the 22th day of gestation, the yolk sac is completely vascularized and is prominent in relation to the size of the canine embryo, so that by mid-gestation the yolk sac is three times larger than the embryo, containing two long reddish projections due to the intensification of vascularization, which is maintained until the moment of birth. At the beginning of gestation, the canine yolk sac is a trilaminar structure composed of an internal epithelium, endothelium and mesenchyme containing blood islets (Aralla et al., 2013). As observed, as pregnancy progresses, there is an increase in the thickness of the mesenchymal layer and in the quantity and size (diameter) of blood islets, suggesting an intensification of vascularization that persists until the end of pregnancy. The more rounded central hemangioblasts play the role of generating the first hemocytopoietic stem cells, while the peripheral ones (irregularly shaped) differentiate into angioblasts, which in turn are the precursors of blood vessels (Mançanares et al., 2013).

To support these previous observations, the analyses showed that the deposition of glucose in the form of glycogen within the cytoplasm of the yolk sac cells is maintained and intensified in the final stages of gestation, reinforcing the role of nutritional reserve played by the canine yolk sac, even in the late stages of gestation. Another characteristic observed during this period of gestation was the abundance of type I collagen around the blood islets, which indicates reinforced structural support, essential for the functional maintenance of the yolk sac at the end of gestation.

In terms of embryology, the prolonged persistence of the yolk sac in carnivores may be related to its conversion from a bilaminar structure to a vascularized trilaminar omphaloplaque after mesodermal invasion of the yolk sac wall. This configuration allows the yolk sac to continue functioning even after its detachment from the trophoblast, in contrast to species whose yolk sac maintains a bilaminar structure, such as marsupials and in the pre-implantation phase of equines (Freyer et al., 2003; Ginther, 2022), leading to earlier regression. Evolutionarily, the formation of a trilaminar omphaloplaque is considered a plesiomorphic condition, possibly present in the common ancestor of eutherians (O’Leary et al., 2013) and preserved in Ferungulata (Mess and Carter, 2006). Furthermore, carnivores exhibit other ancestral reproductive characteristics, such as short gestation periods and highly altricial neonates (Carter and Mess, 2017), which may increase the reliance on persistent and functionally active extraembryonic structures. In this context, the functions of the yolk sac appear to be even more complex than previously thought, also being involved in the biosynthesis of serum proteins before hepatic maturity, acting as an important mediator of exchanges between the embryo and maternal tissues (Tiedemann and Minuth, 1980; Lee et al., 1983).

The structural changes observed in the yolk sac throughout gestation may also be correlated with its hematopoietic activity. Hematopoiesis is a continuous process driven by hematopoietic stem cells (HSCs), which are capable of giving rise to all blood and immune cell lineages (Mosaad, 2014; Dzierzak and Bigas, 2018). In mammals, the yolk sac represents the first hematopoietic site, with blood islets appearing around day 18 of gestation and containing immature hemangioblastic precursors (Mançanares et al., 2013). As gestation advances, hematopoietic activity gradually shifts to the fetal liver and later to the bone marrow (Kruitwagen et al., 2014; Tavares et al., 2020). Although this developmental sequence is well established, little information is available regarding the persistence of these phenotypes at the end of gestation. Fratini et al. (2016) identified OCT4 expression around day 20 of gestation, suggesting the presence of cells with an embryonic-like phenotype in the early yolk sac. In our study, OCT4 immunolabeling remained detectable in blood islets even at late stages of gestation (day 57), indicating that undifferentiated cellular niches persist throughout pregnancy. These findings are in agreement with the study by Wenceslau et al. (2011), who reported the gene expression of pluripotency-associated markers, including OCT4, SOX2, and NANOG, in yolk sac–derived tissues across different gestational stages (25, 30, 40, 45, and 60 days), as assessed by RT-PCR.

## Conclusion

This study presents a detailed morphological and histological characterization of the canine yolk sac during gestation, demonstrating that this extraembryonic membrane remains persistent and functionally active until the end of fetal development. Although the gestational age estimate was indirect due to a lack of reproductive history and the sample size per developmental stage was modest, these limitations are inherent to opportunistic sample collection and should be considered when interpreting the results. Even so, the results provide important data on canine extraembryonic biology and highlight the yolk sac as a multi-functional organ whose importance extends far beyond the beginning of gestation.

## Data Availability

Research data is available in the body of the article.
